# Selenotranscriptome Network in Non-alcoholic Fatty Liver Disease

**DOI:** 10.3389/fnut.2021.744825

**Published:** 2021-11-17

**Authors:** Kaitlin Day, Lucia A. Seale, Ross M. Graham, Barbara R. Cardoso

**Affiliations:** ^1^Department of Nutrition, Dietetics, and Food, Monash University, Notting Hill, VIC, Australia; ^2^Pacific Biosciences Research Center, School of Ocean and Earth Science and Technology, University of Hawaii, Honolulu, HI, United States; ^3^Curtin Medical School, Curtin Health Innovation Research Institute, Curtin University, Perth, WA, Australia

**Keywords:** selenium, non-alcoholic steatohepatitis, selenoproteins, selenocysteine lyase, ferroptosis

## Abstract

Observational studies indicate that selenium may contribute to the pathogenesis of non-alcoholic fatty liver disease (NAFLD). Transcriptomic exploration of the aetiology and progression of NAFLD may offer insight into the role selenium plays in this disease. This study compared gene expression levels of known selenoprotein pathways between individuals with a healthy liver to those with NAFLD. Publicly available gene expression databases were searched for studies that measured global gene expression in liver samples from patients with steatosis and non-alcoholic steatohepatitis (NASH) and healthy controls (with [HOC] or without [HC] obesity). A subset of five selenoprotein-related pathways (164 genes) were assessed in the four datasets included in this analysis. The gene *TXNRD3* was less expressed in both disease groups when compared with HOC. *SCLY* and *SELENOO* were less expressed in NASH when compared with HC. *SELENOM, DIO1, GPX2*, and *GPX3* were highly expressed in NASH when compared to HOC. Disease groups had lower expression of iron-associated transporters and higher expression of ferritin-encoding sub-units, consistent with dysregulation of iron metabolism often observed in NAFLD. Our bioinformatics analysis suggests that the NAFLD liver may have lower selenium levels than a disease-free liver, which may be associated with a disrupted iron metabolism. Our findings indicate that gene expression variation may be associated with the progressive risk of NAFLD.

## Introduction

Non-alcoholic fatty liver disease (NAFLD) is identified as a spectrum of progressive conditions that range from simple accumulation of fat in >5% of hepatocytes (steatosis) to non-alcoholic steatohepatitis (NASH), which is marked by the coexistence of steatosis, liver-cell injury and inflammation (steatohepatitis) (1) and, ultimately, cirrhosis (2). NAFLD is closely linked to obesity and is commonly referred to as the hepatic manifestation of the metabolic syndrome, given that it often coexists with dyslipidaemia, hypertension, insulin resistance and abdominal obesity (2). The prevalence of NAFLD increases proportionally with body mass index (BMI). While the prevalence of NAFLD is about 25% in the general population, it may increase to over 90% amongst individuals with BMI ≥ 30 kg/m^2^ (3).

Oxidative stress plays a pivotal role in the pathogenesis of NAFLD (4). The increase in hepatic iron observed in approximately one-third of NAFLD patients is often considered to play a pathogenic role given iron's ability to donate electrons and catalyse the production of reactive oxygen species (ROS) (5). Thus, it has been proposed that therapies designed to reduce cellular oxidative load may have therapeutic potential for reducing the risk of NAFLD (6, 7). The essential micronutrient selenium was demonstrated to play a pivotal role in redox homeostasis, thus curbing ROS (8). Disturbances in selenium bioavailability or metabolism have been linked with liver diseases such as NAFLD (9). The redox functions of selenium are carried out by selenocysteine (Sec) residues, a cysteine (Cys) cognate with selenium in the place of sulphur, in proteins (10). The human selenoproteome is small, with only 25 selenoproteins identified in the genome (11). Several selenoproteins are designated according to their function: three proteins comprise the iodothyronine deiodinase (DIO) family (DIO1, DIO2, and DIO3) which control thyroid hormone activation; five proteins comprise the glutathione peroxidase (GPX) family (GPX1, GPX2, GPX3, GPX4, and GPX6), involved in detoxification of ROS using glutathione; three proteins comprise the thioredoxin reductase (TRXR) family (expressed from three separate genes: *TXNRD1, TXNRD2*, and *TXNRD3*), which reduces thioredoxin to prevent oxidative damage; methionine sulphoxide reductase B1 (MSRB1), which plays a role in innate immunity by reducing methionine sulphoxide; selenophosphate synthetase 2 (SEPHS2), that provides selenophosphate to the selenoprotein synthesis apparatus; and selenoprotein P (SELENOP), a plasmatic transporter of selenium to other tissues produced in the liver. Other selenoproteins in the human proteome are SELENOF, SELENOH, SELENOI, SELENOK, SELENOM, SELENON, SELENOO, SELENOS, SELENOT, SELENOV, and SELENOW. Most of the selenoproteins act in reactions requiring strong redox capacity, and are deemed critical for several functions in the body, such as immune response, metabolism of thyroid hormones, and detoxification of heavy metals and toxins (12).

Little information is available regarding the link between circulating selenium and NAFLD progression (9). Selenium status has been demonstrated to be lower in individuals with chronic liver diseases such as alcoholic fatty liver (13) and cirrhosis (14). Findings from animal studies suggest a beneficial effect of selenium on NAFLD. Selenium supplementation recovered abnormal liver function tests (15), decreased the number of stellate cells and reduced fibrosis (16) in rodents subjected to liver injury induced by carbon tetrachloride. However, available literature investigating the role of selenium in human NAFLD is limited to observational studies, which suggest that selenium, particularly *via* the selenium transporter, SELENOP, may play a detrimental role in NAFLD pathogenesis (9). High selenium dietary intake was associated with increased risk of NAFLD in a Chinese population (17). A cross-sectional epidemiological study in Chinese adults reported that those in the highest quartile for plasma selenium (>247.4 μg/L) had a 54% higher prevalence of NAFLD compared with those in the lowest quartile (<181.6 μg/L) (18). Choi et al. (19) reported that adults in the highest tertile for plasma SELENOP had a 7.5-fold greater risk of NAFLD than those in the lowest tertile. Similarly, a cross-sectional study demonstrated that serum SELENOP was 6.8-fold higher in adults with NAFLD when compared with healthy controls (20). Furthermore, SELENOP has been associated with severity of NAFLD in two cross-sectional studies involving patients in China and Italy (21, 22), however it should be noted that in some of these studies additional biomarkers of total selenium were not assessed, lacking thus a more comprehensive profile of selenium in NAFLD. As only observational studies have been conducted to investigate the association between selenium and NAFLD beyond SELENOP, studies that provide mechanistic insights are necessary to elucidate the role of selenium in the pathogenesis of NAFLD.

Recently, particular attention has been directed to exploring a gene expression signature of NAFLD to understand the molecular pathways that underpin NAFLD aetiology and disease progression (23–26). Due to the global and exploratory nature of these studies, particular pivotal pathways and their associated genes have not been explored. Notably, pathways involving selenoproteins and selenium metabolism have not been specifically investigated. Hepatic selenium and selenoprotein-related pathways comprise genes involved in the metabolism of selenium and selenoproteins, interaction of selenoproteins with other micronutrients, antioxidant response and ferroptosis. These pathways are of great interest as they can elucidate how selenium underlies chronic pathological mechanisms in NAFLD. Using publically available global gene expression datasets, this study aimed to compare gene expression of known selenoprotein and selenium-containing pathways between individuals with healthy liver to those with steatosis and NASH. Given that individuals with obesity present with a higher risk of NAFLD than lean individuals, we further stratified healthy individuals into obese and non-obese for a more informative assessment.

## Materials and Methods

**Figure 1:**
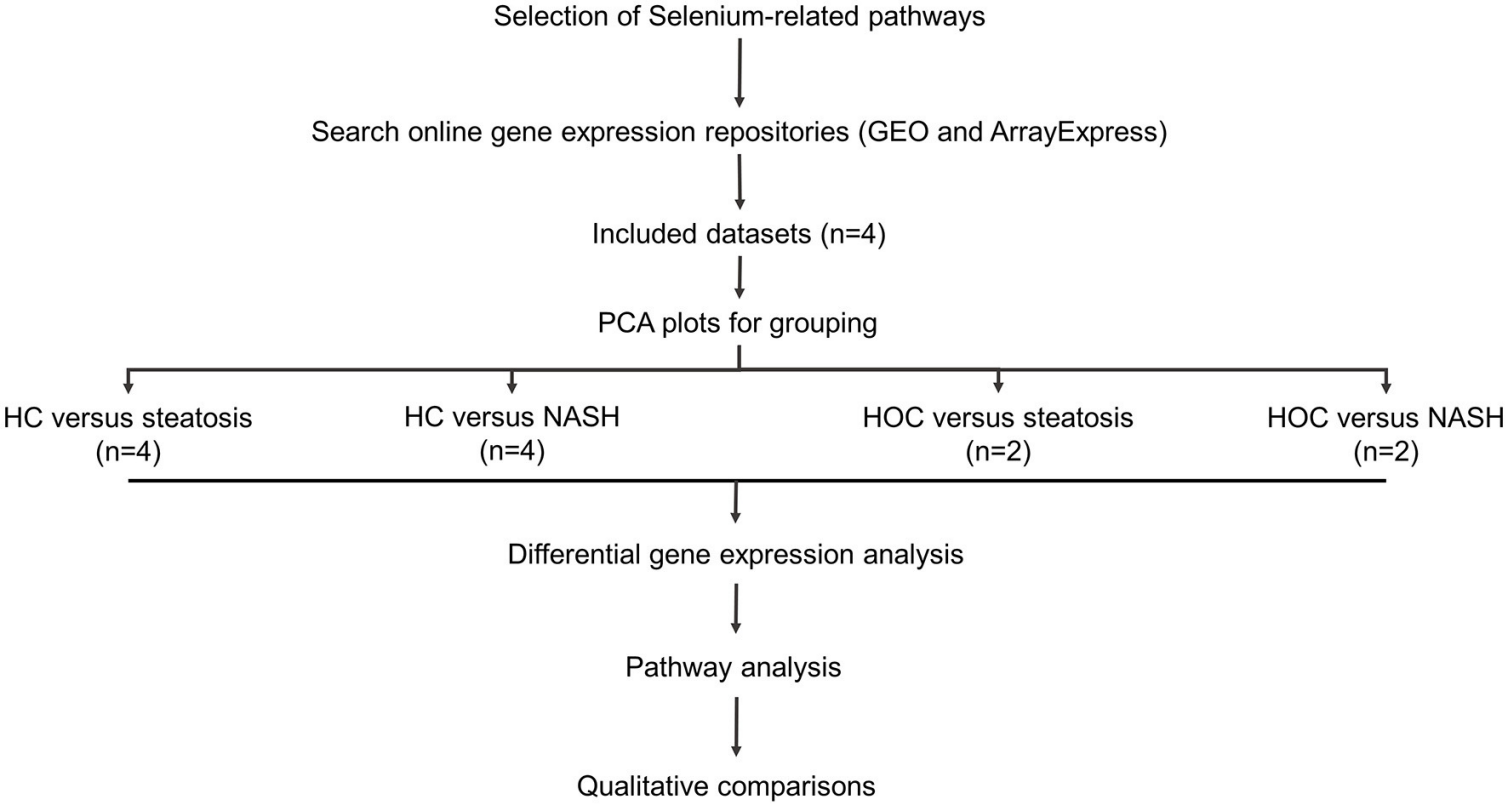
Study workflow. Included datasets were downloaded from GEO or ArrayExpress. PCA plots were used to determine whether normal-weight healthy control (HC) and healthy obese controls (HOC) could be grouped together and whether steatosis and NASH patients could be grouped. All groups were analysed individually. Differentially expressed genes were defined as genes with an adjusted *P*-value < 0.05. For pathway analysis, genes of interest were defined as genes with a log fold change in the same direction in the majority of datasets (HC: 3/4 datasets, HOC: 2/2 datasets) and where the difference was significant in at least 50% of these datasets. HC, normal-weight healthy controls; HOC, healthy obese controls; NAFLD, non-alcoholic fatty liver disease. n, number of studies used for the analysis.

Figure 1 provides an overview of the methods used in this study.

### Dataset Selection

Gene expression datasets from online repositories (GEO and ArrayExpress) were selected based on the following criteria: (i) contained liver biopsy samples from healthy individuals (with or without obesity) and from individuals with a clinical diagnosis of steatosis or NASH; and (ii) raw gene expression data was available for download and re-analysis for all samples. Both microarray and RNA sequencing data were included. Demographics (age, sex, BMI, and disease state) were gathered from the papers associated with each dataset. Where there were discrepancies in numbers between datasets and their corresponding published papers, the demographics reported in the dataset description files were used, if available. When this information was not available, the differences in numbers are reported. Subjects from all the datasets provided written informed consent before tissue sample donation and inclusion in the respective studies.

### Pathway Selection

Five pathways from the Wikipathways database (27) were identified as having selenium or selenoproteins as key elements. The pathways were: Selenium metabolism and selenoproteins (WP28); Selenium micronutrient network (WP15); Ferroptosis (WP4313); Glutathione metabolism (WP100); and Oxidative stress (WP408). The ferroptosis pathway was included because the selenoprotein GPX4 is a key regulator of this cell death pathway. These pathways contained a total of 164 unique genes. Gene identifiers were extracted from chosen pathways and used to create a subset of the normalised gene expression data for each dataset. These subsets were used for downstream analysis and data visualisation.

### Pre-processing and Normalisation

All pre-processing and statistical analysis was performed in R (version 3.6.3). Pre-processing and normalisation of microarray data was performed using the ArrayAnalysis pipeline that has been previously described (28). ArrayAnalysis utilises the functions within the affy package (version 1.62) for background correction, and robust multi-array normalisation was applied to all microarray datasets. Raw gene counts were downloaded from GEO and were used for all downstream analysis of RNA sequencing data.

### Statistical Analysis

All differential gene expression analysis was conducted within studies. Principle component analysis (PCA) plots were used to assess the degree of overlap in expression data between control (normal weight and healthy obese) and disease (steatosis and NASH) groups. For microarray data, these plots were generated using the “prcomp” functions in the R base stats package (version 3.6.3), and graphs generated using the “factoextra” (version 1.0.7) and “ggplot2” packages (version 3.3.3) in R. For RNA sequencing data, the “plotPCA” function within the DESeq2 package (version 1.24.0) was used to generate PCA plots. Linear mixed modelling was utilised to assess significantly differentially expressed genes in each comparison. For microarray data, linear mixed modelling was completed using the “limma” package (version 3.40.6) in R and for RNA sequencing data, the “DESeq2” package was used. Log fold changes were calculated as log base 2 of the fold change.

Benjamini-Hochberg adjustment for multiple testing was applied at the gene level to determine significance for genes differentially expressed between groups. Significance of differential gene expression was inferred at an adjusted *P*-value < 0.05.

Comparisons across studies were made at the pathway level. In this way, genes of interest were defined as those in which (i) the log fold change between groups was in the same direction in the majority of studies and (ii) this reached significance (adjusted *P*-value < 0.05) in at least 50% of these studies.

### Data Visualisation

Differential gene expression data (log fold changes and adjusted *P*-values) for all the included datasets were visualised on chosen pathways using PathVisio (Version 3.3.0). Overlap of significant differentially expressed genes between studies was visualised using Euler or Venn diagrams as appropriate.

## Results

### Study Characteristics

Out of 11 originally identified datasets, five met the inclusion criteria (GSE126848, E-MEXP-3291, GSE61260, GSE63067, and GSE48452) (25, 29–32). The remaining six were excluded as they either did not have a healthy control group or raw data was not available. Two of the included datasets (31, 32) pertained to the same cohort (GSE61260 and GSE48452) and contained overlapping samples, hence only the dataset which contained the highest number of samples (GSE61260) (31) was included in the analysis, to prevent over-sampling. All four datasets included in this analysis contained samples from subjects diagnosed with steatosis and NASH. Two datasets (29, 31) contained samples from HC and HOC, and the other two datasets contained only HC samples (25, 30). Control samples from the included studies were obtained from: (i) cholecystectomy surgery (25), (ii) healthy individuals recruited at the hospital (no further details on tissue collection were provided) (29), (iii) major oncological surgery where liver malignancy was excluded (31), or (iv) liver tissue bank (30). Subjects from all the datasets provided written informed consent before tissue sample donation and inclusion in the respective studies.

Table 1 describes the study characteristics of the four public datasets included in our analysis. Suppli et al. (29) have only included samples from males without diabetes, while the other studies did not have such a restrictive selection criterion. However, Frades et al. (25) and Lake et al. (30) did not provide information about diabetes status. BMI, reported by all the studies except Lake et al. (30), was notably lower in the population included in the study by Suppli et al. (29) than the other studies. Where BMI was reported, it was adequate (18.5–24.99 kg/m^2^) in all HC groups. Histopathological evaluation of samples was performed using different scoring systems among the studies: Suppli et al. (29) used the Steatosis-Activity-Fibrosis (SAF) algorithm (33); Horvath et al. (31) and Lake et al. (30) used the NAFLD activity score (NAS) (34); and Frades et al. (25) used the semiquantitative evaluation method proposed by Brunt et al. (35).

**Table 1:** Study characteristics.

**Study, country**	**Study population**	**Dataset**	**NAFLD diagnosis**	**Study groups**	**Groups characteristics**	**Method**
Suppli et al. (29), Denmark	Adults without diabetes and absence of excessive alcohol intake (>20/12 g/day for male/female)	GSE126848	Ultrasonographic evidence of hepatic steatosis, elevated liver enzymes, and compatible liver histology^a^ in the absence of other (viral, alcohol, metabolic) causes of steatohepatitis.	Normal-weight healthy controls (BMI: 18.5–25) (*n* = 14/14)	Age: 39.5 ± 12.0 Male: 100% BMI: 23.1 ± 1.6 No lobular inflammation, hepatocyte, ballooning, or fibrosis 1 individual showed steatosis (stage 1)	RNA-Seq
				Healthy obese controls (BMI: 30–40 kg/m^2^) (*n* = 12/12)	Age: 36.6 ± 10.2 Male: 100% BMI: 33.2 ± 1.3 No lobular inflammation, hepatocyte, ballooning, or fibrosis 1 individual showed steatosis (stage 1)	
				Steatosis: hepatic steatosis, elevated liver enzymes, compatible liver histology; presence of steatosis in >5% of hepatocytes; SAF <2^a^ (*n* = 15/15)	Age: 39.4 ± 10.6 Male: 60% BMI: 32.8 ± 5.1 Steatosis (mostly severe) Mild-grade lobular inflammation	
				NASH: presence of steatosis in > 5% of hepatocytes; SAF ≥ 2^a^; hepatocyte ballooning morphology (*n* = X16/16)	Age: 38.9 ± 17.0 Male: 75% BMI: 33.9 ± 6.2 Fibrosis stage: 1–2	
Horvath et al. (31), Germany	Liver samples obtained from adults during major oncological surgery where liver malignancy was excluded (control group); liver samples from adults with suspected NAFLD undergoing liver biopsy or assessment of liver histology (NAFLD groups)	GSE61260	Histological evidence according to NAFLD activity score (NAS)^b^ in the absence of other (viral, alcohol, metabolic) causes of steatohepatitis	Normal-weight healthy controls (*n* = 38/38)	Age: 58.1 ± 18.3 Male: 42% BMI: 23.9 ± 3.0	Microarray: Affymetrix Human Gene 1.1 ST Array
				Healthy obese controls (*n* = 24/24)	Age: 49.1 ± 11.4 Male: 21% BMI: 42.9 ± 8.0	
				Steatosis (*n* = 23/23)	Age: 41.3 ± 6.2 Male: 52% BMI: 51.1 ±9.3	
				NASH (*n* = 24/24)	Age: 45.3 ±12.3 Male: 50% BMI: 51.2 ±10.8	
Frades et al. (25), Spain	Liver samples from adults obtained during laparoscopic cholecystectomy (control group) or laparoscopic bariatric surgery (NAFLD groups).	GSE63067	Histological evidence (macrovesicular steatosis, lobular and portal inflammation)^c^ in the absence of other (viral, alcohol, metabolic) causes of steatohepatitis.	Normal-weight healthy controls (*n* = 7/6)	Age: 57.6 (23–79 y) Male: 50% BMI: 24.6 Diabetes: NA	Microarray: Affymetrix Human Genome U133 Plus 2.0 Array
				Steatosis (*n* = 2/6)	Age: 43.3 (23–72 y) Male: 67% BMI: 48.2 Diabetes: NA	
				NASH: steatohepatitis grade 1 (macrovesicular steatosis, lobular and portal inflammation) (*n* = 9/9)	Age: 41.1 (24–61 y) Male: 78% BMI: 44.4 Diabetes: NA	
Lake et al. (30), United States	Liver samples acquired from liver tissue bank	E_MEXP_3291	Histological evidence (macrovesicular steatosis, lobular and portal inflammation)^b^ in the absence of other (viral, alcohol, metabolic) causes of steatohepatitis.	Normal-weight healthy controls (*n* = 19/19)	Age: 42.15 (16–70 y) Male: 55% BMI: NA Diabetes: NA	Microarray:Affymetrix GeneChip Human 1.0ST Arrays
				Steatosis: >10% fat deposition without inflammation or fibrosis (*n* = 10/10)	Age: 46.7 (16–66 y) Male: 40% BMI: NA Diabetes: NA	
				NASH with fatty liver: >5% fat deposition, marked inflammation, fibrosis (*n* = 9/9)	Age: 56.8 (41–68 y) Male: 11% Diabetes: NA	
				NASH without fatty liver: <5% fat deposition, increased inflammation, fibrosis (*n* = 7/7)	Age: 52.7 (33–66 y) Male: 11% BMI: NA Diabetes: NA	

*Data presented as mean ± SD or mean (range). n = samples included in this analysis/samples included in the original study*.

a *According to Steatosis-Activity-Fibrosis (SAF) (33)*.

b *According to NAFLD activity score (NAS) (34)*.

c *According to Brunt et al. (35)*.

*BMI, Body Mass Index; NAFLD, non-alcoholic fatty liver disease; NASH, non-alcoholic steatohepatitis*.

Functional analysis was reported in two of the original studies: out of the 16 gene ontology (GO) terms identified by Ahrens et al. (32) [which contains a large subset of gene expression data reported by Horvath et al. (31)], 12 terms were also identified when we used our list containing the 164 selected genes. Furthermore, our list of genes resulted in nine KEGG (Kyoto Encyclopaedia of Genes and Genomes) pathways similar to those identified by Frades et al. (25). See Supplementary Table 5 for this full list.

### PCA Plots

Groups within studies were compared to assess whether control [Normal weight healthy control (HC) and Healthy obese control (HOC) groups] and/or disease (steatosis and NASH groups) samples within studies could be pooled for differential analysis. Overall, PCA plots revealed that controls were different within studies and, as such, these groups were kept separate. Regarding steatosis and NASH samples, PCA plots for Suppli et al. (29) and Horvath et al. (31) revealed that steatosis and NASH samples were not different, while the plots for Lake et al. (30) indicated that the two groups were different. Inconclusive information could be extracted from Frades et al. (25) given the limited sample size (*n* = 2) for steatosis. Despite the similarities observed for two studies, steatosis and NASH were kept separate for analysis as they have clearly distinct clinical phenotypes (Supplementary Figure 1).

### Differentially Expressed Genes Within Studies

Of the 164 genes included in this analysis, four genes (*UGT1A6, MAPK10, INS*, and *TSTD1*) had no gene expression data available in any of the included studies.

#### Normal-Weight Healthy Controls vs. Steatosis

**Figure 2:**
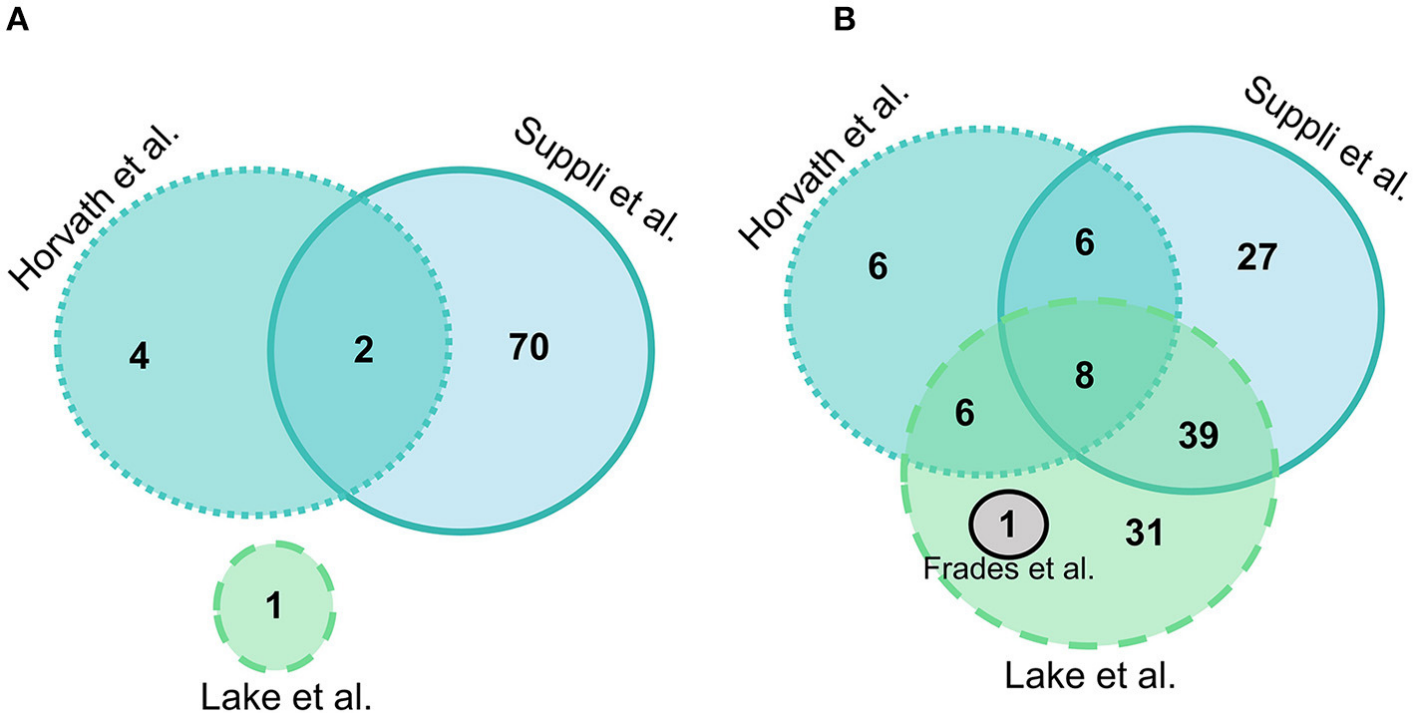
Euler diagram of the number of significantly differentially expressed genes (adjusted *P*-value < 0.05) in liver samples: **(A)** Normal-weight controls vs. steatosis; **(B)** Normal-weight controls vs. NASH. Grey indicates Frades et al. (25), light blue indicates Suppli et al. (29), green indicates Lake et al. (30), aqua indicates Horvath et al. (31).

Suppli et al. (29) had the most differentially expressed genes between HC and steatosis groups with 72 genes presenting statistically significant differences, followed by Horvath et al. (31) with 6, Lake et al. (30) with 1, and Frades et al. (25) had no genes significantly different between groups. A total of two genes were common in two out of four datasets (*MTR* and *GSTO1*), but no gene was differentially expressed between HC and steatosis in all datasets (Figure 2A). A list with all the genes included in the comparison between HC and steatosis across the datasets is available in Supplementary Table 1.

Suppli et al. (29) was the only study that demonstrated differentially expressed selenoprotein genes between HC and steatosis groups. Amongst the 13 genes, eight (*DIO1, GPX2, SEPHS2, SELENOK, SELENOS, SELENOT, SELENOF*, and *SELENOW*) had higher, and five (*DIO3, GPX1, SELENON, SELENOO*, and *TXNRD3*) had lower expression in steatosis.

#### Normal-Weight Healthy Controls vs. NASH

Overall, there were more genes significantly different between HC and NASH than in the HC vs. steatosis comparison. In HC vs. NASH comparison, Lake et al. (30) had the most genes significantly differentially expressed with 86 genes, followed by Suppli et al. (29) with 80 genes, Horvath et al. (31) with 27 and Frades et al. (25) with 1 gene. A total of eight genes were common in three out of four datasets (*PLG, JUN, ACSL4, MTR, CBS, GGT5, SLC11A2*, and *SOD1*), but no gene was differentially expressed between HC and NASH in all datasets (Figure 2B; Supplementary Table 2).

In Lake et al. (30) all the 14 selenoprotein genes differentially expressed between the two groups (*SELENOF, SELENOI, SELENOK, SELENOO, SELENOS, SELENOV, SELENOT, SEPHS2, TXNRD2, TXNRD3, GPX1, GPX2, MRSB1*, and *DIO2*) had a lower expression in NASH. In Suppli et al. (29), all the genes with higher expression in steatosis were also more expressed in NASH when compared to HC (*SELENOF, SELENOK, SELENOS, SELENOT, SELENOW, GPX2, GPX3, DIO1*, and *SEPHS2*), in addition to the *TXNRD1* gene. Similarly, four out the five genes with lower expression in steatosis also had lower expression in NASH (*SELENOO, SELENON, DIO3*, and *TXNRD3*) compared to HC. Finally, Horvath et al. (31) had four selenoprotein genes differentially expressed between HC and NASH, one with lower expression (*SELENOP*) and three with higher expression (*SELENON, GPX1*, and *GPX4*) in NASH. No significant differences were observed for selenoprotein genes between HC and NASH groups in Frades et al. (Supplementary Table 2). The *SCLY* gene, which encodes a key enzyme in selenium metabolism, was less expressed in NASH in Lake et al. (30) and Frades et al. (25).

#### Healthy Obese Controls vs. Steatosis

Two studies had HOC data available for comparison with steatosis and NASH. Overall, there were fewer genes significantly different between HOC and both steatosis and NASH samples. Suppli et al. (29) was the only study out of the two that demonstrated differentially expressed genes between HOC and steatosis with 62 genes (30 with lower expression and 32 with higher expression in steatosis). Of these genes, 11 coded for selenoproteins of which four genes had lower expression (*SELENON, SELENOO, GPX1*, and *TXNRD3*) and seven genes had higher expression (*SELENOK, SELENOS, SELENOW, GPX2, GXP3, DIO1*, and *SEPHS2*) in steatosis. The log fold changes of all genes were in the same direction as the comparison between HC vs. steatosis and NASH (Supplementary Table 3).

#### Healthy Obese Controls vs. NASH

**Figure 3:**
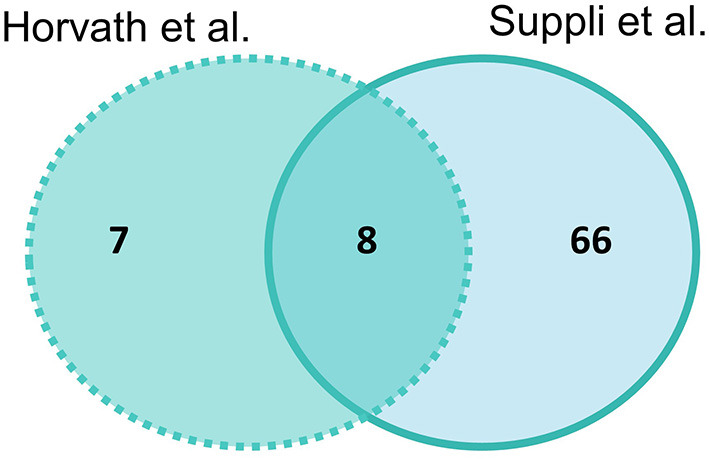
Venn diagram of number of significantly differentially expressed genes (adjusted *P*-value < 0.05) in liver samples of healthy obese controls vs. NASH. Light blue indicates Suppli et al. (29), aqua indicates Horvath et al. (31).

Suppli et al. (29) had the most differentially expressed genes for HOC vs. NASH with 74 genes and Horvath et al. (31) had 15 genes, with eight genes overlapping between studies (*SELENON, TNXRD3, PLG, MAOA, SAT2, ALOX5, GGT5*, and *ACSL4*) (Figure 3). Of these overlapping genes, three genes were less expressed (*PLG, TXNRD3*, and *MAOA*) and two (*GGT5* and *ACSL4*) more expressed in NASH in both studies. The log-fold changes of the other three genes were in opposite directions between studies: *SAT2* had higher expression in NASH in Suppli et al. (29) and lower expression in Horvath et al. (31); and *ALOX5* and *SELENON* had lower expression in NASH in Suppli et al. (29) and higher expression in Horvath et al. (31) (Supplementary Table 4).

### Pathway Analysis

#### Normal-Weight Healthy Controls vs. Disease Groups (Steatosis and NASH)

**Figure 4:**
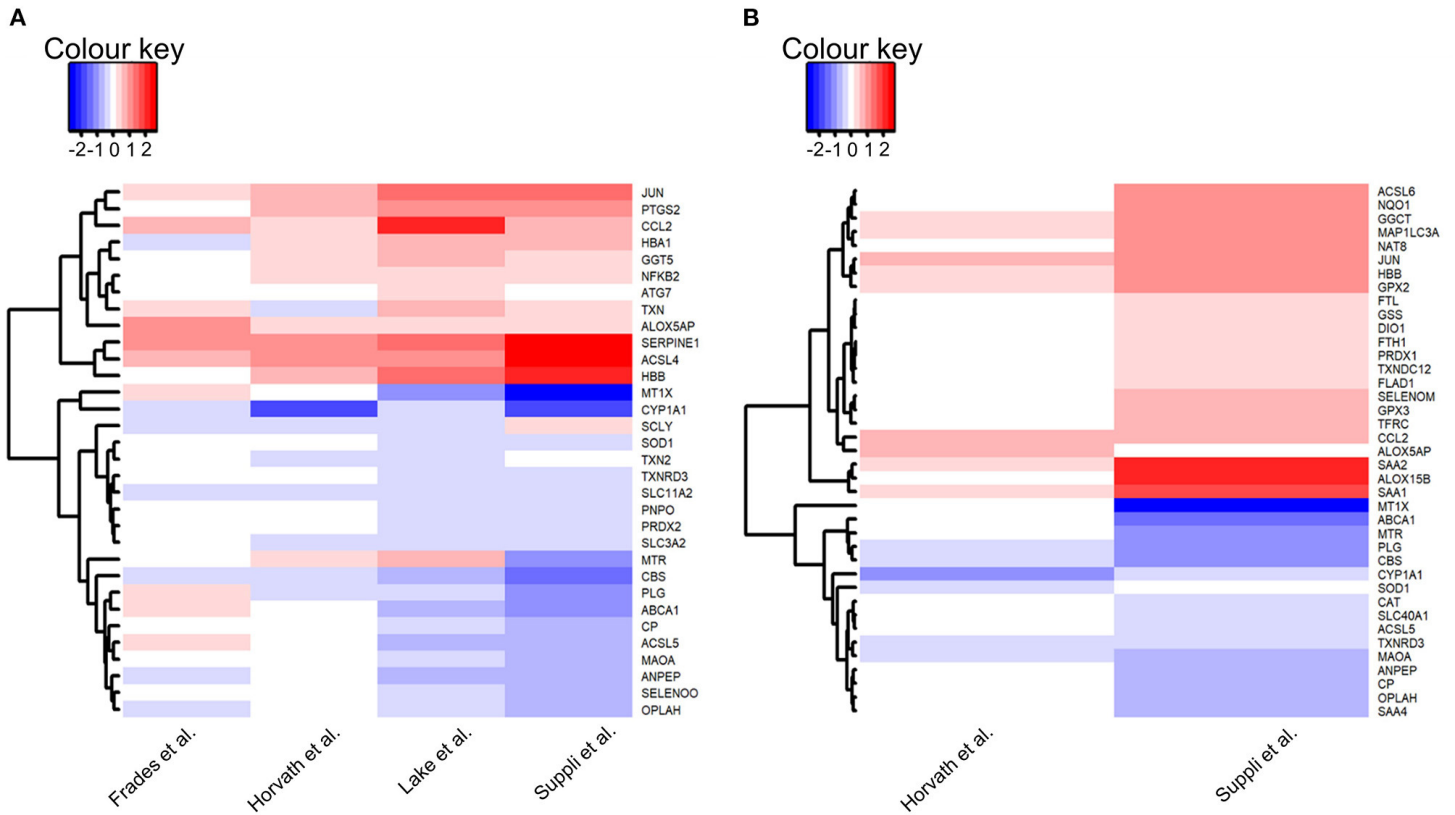
Heatmaps of genes of interest in liver samples: **(A)** Normal-weight healthy controls vs. NASH; **(B)** Healthy obese controls vs. NASH. Genes of interest were defined as genes for which the log fold change was in the same direction in the majority of datasets (HC: 3/4 datasets, HOC: 2/2 datasets) and with an adjusted *P*-value < 0.05 in at least 50% of these datasets. Red depicts increased expression in NASH compared to controls and blue depicts decreased expression in NASH compared to controls. Heatmaps were generated using the heatmap.2 function in gplots (version 3.0.3) in R (version 3.6.3).

Within the explored pathways, genes were considered of interest if the log fold change between HC and disease groups were in the same direction in three out of four included datasets, and the difference was significant (adj. *P*-value < 0.05) in at least two of the three datasets. There were no genes that met these criteria when HC was compared with steatosis, and therefore no pathways were explored. A total of 32 genes were of interest in the comparison HC vs. NASH (Figure 4A).

Of the five pathways explored in this analysis, no pathway was consistently more highly or lowly expressed in NASH compared to HC. There were no consistent differences in expression for selenoprotein genes between groups in the “glutathione metabolism” and “ferroptosis” pathways. In the “glutathione metabolism” pathway, expression of two genes was lower in NASH (*OPLAH* and *ANPEP*) and expression of *GGTA1* was higher. In the “ferroptosis” pathway, four genes (*CP, SLC3A2, SLC11A2*, and *ACSL5*) had lower expression in NASH and two genes (*ATG7* and *ACSL4*) had higher expression.

There were no consistent differences in gene expression of selenoproteins across studies between NASH and HC samples in the “oxidative stress” pathway. However, there was a significantly lower expression of repression of ROS producing systems, notably, a decrease in *CYP1A1* expression in NASH.

In the “selenium metabolism and selenoproteins” pathway, the selenoproteins *TXNRD3* and *SELENOO*, as well as the gene *SCLY*, were less expressed in NASH while the gene *JUN* was more expressed in the disease group. There were no differences in selenoprotein genes in the “selenium micronutrient network” pathway, which encapsulates several genes encompassed in the other pathways. Nonetheless, nine genes (*TXN, ALOX5AP, HBA1, HBB, COX2, NF*κ*B, MTR, CCL2*, and *SERPINE1*) had higher expression in NASH whereas six genes, including the already mentioned *TXNRD3* and *SELENOO* as well as *SOD1, PNPO, CBS*, and *PLG*, had lower expression in NASH.

A summary of all the genes of interest differently expressed between HC vs. disease groups according to the examined pathways is presented in Table 2.

**Table 2:** Genes of interest in the comparisons normal-weight healthy controls (HC) × steatosis, normal-weight healthy controls (HC) × NASH, healthy obese controls (HOC) × steatosis, healthy obese controls (HOC) × NASH.

**Pathways**	**HC × steatosis**	**HC × NASH**	**HOC × steatosis**	**HOC × NASH**
Glutathione metabolism	◯	↓ *OPLAH* and *ANPEP* ↑ *GGTA1*	↓ *OPLAH* ↑ *GPX2* and *GPX3*	↓ *OPLAH* and *ANPEP* ↑ *GSS* and *GGTLA1* ↑ *GPX2* and *GPX3*
Ferroptosis	◯	↓*CP, SLC3A2, SLC11A2*, and *ACSL5* ↑ *ATG7* and *ACSL4*	↓ *SLC40A1* and *MAP1LC3C* ↑ *FTL, ATG5*, and *MAP1LC3A*	↓ *SLC40A1, CP*, and *MAP1LC3C* ↑ *FTH1, FTL, ACSL6, ACSL4, MAP1LC3A, TFRC*, and *GSS*
Oxidative stress	◯	↓ *CYP1A1*	↓ *NFκB* and *MXT1*	↓ *MAOA, CYP1A1*, and *MT1X*
Selenium metabolism and selenoproteins	◯	↓ *TXNRD3* and *SELENOO* ↓ *SCLY* ↑ *JUN*	↓ *TXNRD3* ↑ *GPX2* and *DIO1* ↑ *SEPHS2* and *TRNAU1AP*	↓*TXNRD3* ↑ *GPX2, GPX3*, and *DIO1* ↑ *JUN*
Selenium micronutrient network	◯	↓ *TXNRD3* and *SELENOO* ↓ *SOD1, PNPO, CBS*, and *PLG* ↑ *TXN, ALOX5AP, HBA1, HBB, COX2, NFκB, MTR, CCL2*, and *SERPINE1*	↓ *ABCA1, MTR, MTHFR*, and *PLG* ↑ *DIO1* ↑ *HBB, SAA1, SAA2*, and *SEPHS2*	↓ *SOD1, SAA4, ABAC1, MTR, CBS*, and *PLG* ↓ *TXNRD3* ↑ *SAA1, SAA2, CCL2, HBB*, and *SERPINE1* ↑ *SELENOM, DIO1, GPX2*, and *GPX3*

↓ *Lower expression in the disease group (either steatosis or NASH); ↑ higher expression in the disease group (either steatosis or NASH). HC, Normal-weight Healthy Controls; HOC, Healthy Obese Controls. Full gene names are available in Supplementary Tables 1–4. ◯ Means no genes of interest were found in this comparison (HC vs steatosis)*.

#### Healthy Obese Controls vs. Disease Groups (Steatosis and NASH)

##### HOC vs. Steatosis

Genes were considered of interest in the assessed pathways if the log fold change in the comparison of HOC vs. steatosis or NASH was in the same direction in both included datasets and the difference was significant (adj. *P*-value < 0.05) in at least one dataset. Twenty-one genes met these criteria in HOC vs. steatosis and 39 in HOC vs. NASH comparison (Figure 4B).

For the “ferroptosis” pathway three genes were higher (*FTL, ATG5*, and *MAP1C3A*) and two were lower in steatosis (*SLC40A1* and *MAP1LC3C*). Two selenoprotein genes (*GPX2* and *GPX3*) encompassed in the “glutathione metabolism” pathway had higher expression, and one gene had lower expression (*OPLAH*) in steatosis, suggesting an increase in glutathione utilisation. For the “oxidative stress” pathway, two genes (*NF*κ*B1* and *MTX1*) were lower in steatosis compared to HOC. Besides the selenoproteins *GPX2, DIO1*, and *SEPHS2*, the gene for *TRNAU1AP* also had higher expression in steatosis in the “selenium metabolism and selenoproteins” pathway, and *TXNRD3* had lower expression in steatosis. Four genes had lower expression in steatosis (*ABCA1, MTR, MTHFR*, and *PLG*) and five genes had higher expression (*SEPHS2, DIO1, HBB, SAA1*, and *SAA2*) in the “selenoprotein micronutrient network” pathway.

##### HOC vs. NASH

For the “ferroptosis” pathway, expression of seven genes was higher (*FTH1, FTL, ASCL6, ACSL4, MAP1LC3A, TFRC*, and *GSS*) and expression of three genes was lower (*SLC40A1, CP*, and *MAP1LC3C*) in NASH. Expression of *SLC40A1*, the gene encoding the iron exporter ferroportin, was lower in NASH. Interestingly, expression of *MAP1LC3C*, a paralogue of *MAP1LC3A*, was also lower in the disease group. However, the expression of *MAP1LC3C* is not high in the liver (36), so this observation may not be biologically significant. In addition to *GSS*, three genes encompassed in the “glutathione metabolism” pathway had higher expression (*GGTLA1, GPX2*, and *GPX3*), and two genes had lower expression (*OPLAH* and *ANPEP*) in NASH, suggesting an increase in glutathione utilisation. For the “oxidative stress” pathway three genes (*MAOA, CYP1A1*, and *MT1X*) had lower expression in NASH.

For the “selenium metabolism and selenoproteins” pathway, in addition to the genes already mentioned (*GPX2* and *GPX3*), expression of *DIO1* and *JUN* was higher, and *TXNRD3* was lower in NASH. Finally, in the “selenium micronutrient network” pathway, 11 genes (*SELENOM, DIO1, GPX2, GPX3, ALOX15B, FLAP, SAA1, SAA2, CCL2, HBB*, and *SERPINE1*) had higher expression in NASH and seven genes (*TXNRD3, SOD1, SAA4, ABCA1, MTR, CBS*, and *PLG*) had lower expression in NASH compared to HOC.

A summary of all the genes of interest differentially expressed between HC vs. disease groups according to the assessed pathways is presented in Table 2.

### Comparisons Between HC vs. NASH and HOC vs. NASH

There were 17 genes of interest commonly different in both the HC vs. NASH and HOC vs. NASH comparisons. Of these 17 genes, 12 had lower expression in NASH compared to both HC and HOC and four had higher expression in NASH. One gene, *MTR*, had higher expression in NASH compared to HC but lower expression in NASH compared to HOC (Table 2).

## Discussion

**Figure 5:**
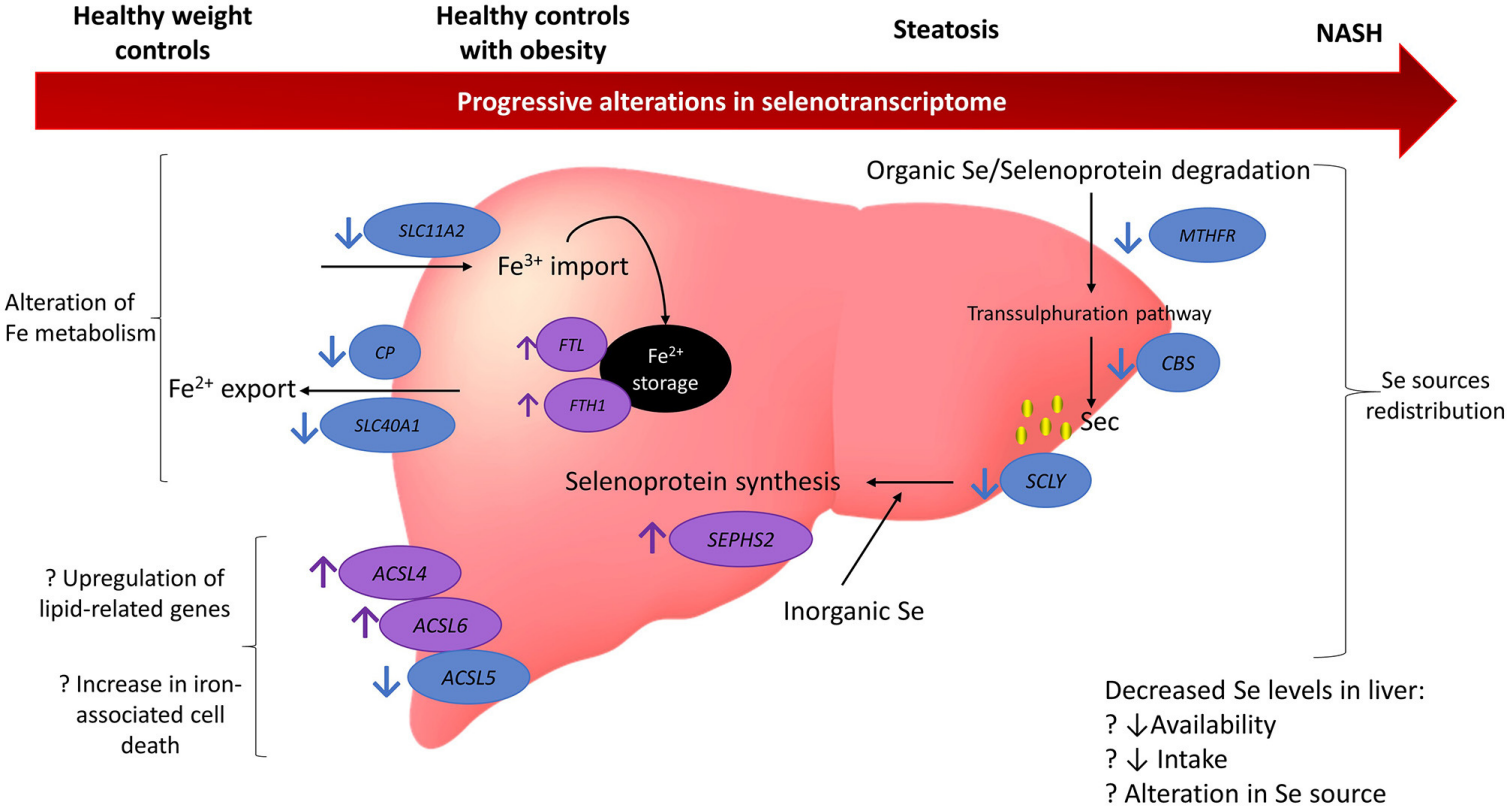
Model summarising the findings from gene expression analysis of 164 genes related to selenium and selenoproteins. Findings suggest there may be a redistribution in selenium sources with a reduction in organic selenium degradation *via* the transsulphuration pathway and an increase in the metabolism of inorganic selenium. Iron metabolism may also be disturbed with an increase in iron storage and promotion of factors related to iron-associated cell death. There may be several reasons for these alterations and they appear to be progressively altered as NAFLD deteriorates. Blue circles and arrows indicate genes down-regulated in NAFLD compared to controls (either healthy weight or obesity) and the purple circles and arrows indicate genes up-regulated in NAFLD compared to controls. Question marks indicate hypothesised reasons for alterations that cannot be fully tested in the current study. MTHFR, methylenetetrahydrofolate reductase; CBS, cystathionine beta-synthase; SCLY, selenocysteine lyase; ACSL5, acyl-CoA synthetase long chain family member 5; ACSL6, acyl-CoA synthetase long chain family member 6; ACSL4, acyl-CoA synthetase long chain family member 4; SCL40A1, solute carrier family 40 member 1; CP, ceruloplasmin; SLC11A2, solute carrier family 11 member 2; Fe, iron; NASH, non-alchoholic steatohepatitis; Se, selenium.

This study used publicly available human global gene expression datasets to uncover pathways involving selenium and selenoproteins that may participate in chronic pathological mechanisms of NAFLD (Figure 5). Previous observational studies have suggested an association between selenium and SELENOP levels in plasma with increased risk and severity of NAFLD (18–22). Nonetheless, the bioinformatics approach undertaken in this study revealed no differences at the gene level for *SELENOP*. Despite this result, it is possible that SELENOP quantities, as well as of other selenoproteins, are still elevated in NAFLD due to regulation of their translation at the ribosome level (37, 38), downstream of transcriptional mechanisms evaluated herein. Furthermore, we observed that the gene encoding the selenoprotein *TXNRD3* was less expressed in both disease groups (NASH and in steatosis) when compared with HOC, while no significant differences between HC and steatosis samples were observed. Also, *SCLY*, which encodes the enzyme responsible for catalysing removal of selenium from selenocysteine, was less expressed in NASH when compared with HC, as was the selenoprotein *SELENOO*. In contrast, gene expression of the selenoproteins *SELENOM, DIO1, GPX2*, and *GPX3* was higher in the NASH group when compared to HOC. Moreover, lower expression of iron-associated transporters, higher expression of genes encoding the ferritin subunits, as well as differential expression of members of the *ACSL* gene family, point to a disturbed iron metabolism which may enhance ferroptosis under conditions of NAFLD. We also observed that for several genes, the difference between NASH and HC was larger than when NASH was compared with HOC or steatosis, supporting the notion that gene expression variation may be associated with the progressive risk of NAFLD.

Although we here present a novel approach that targeted specific molecular pathways, we note that some of these pathways have been previously described as to be perturbed across increasing NAFLD severity in previous studies. For instance, Hoang et al. (39) reported that pathways for glutathione metabolism (“glutathione synthesis and recycling”) and oxidative stress (“oxidative stress induced senescence”) were upregulated in liver samples as the NAS and fibrosis stage increased, although no associations were observed for selenium or selenoprotein pathways. Further, Zhu et al. (40) identified several upregulated selenoproteins (*SELENON, SELENOP, SELENOT, SELENOW, DIO2, DIO3, GPX4*, and *GPX5*) in mild NAFLD liver samples when compared to healthy controls. This therefore suggests that selenium related processes are progressively perturbed in NAFLD development and that a targeted approach as taken by the current study can be used to uncover the specific pivotal pathways and genes involved.

The essential role of selenoproteins in hepatic function was highlighted after mice genetically devoid of hepatic selenoproteins developed hepatocellular degeneration and necrosis, leading to early death (41). Selective loss of housekeeping selenoproteins, such as DIO1, SELENOP, TXNRD1, and SELENOF, led to upregulation of genes involved in cholesterol biosynthesis, and downregulation of genes involved in cholesterol metabolism and transport, suggesting an overall effect of this subset of selenoproteins in hepatic lipoprotein metabolism that favours hypercholesterolaemia (42). Amongst the selenoproteins associated with the metabolism of cholesterol, our findings revealed a higher expression of *DIO1* in the disease groups (NASH and steatosis) when compared to healthy obese controls. Lower expression of the selenoprotein *TXNRD3* was observed in the disease groups when compared with both control groups (HC and HOC). The isoform 3 for TRXR, encoded by *TXNRD3*, has an atypical kinetic behaviour when compared to the other two TRXR isoforms as it can reduce both thioredoxin and glutathione, and thus can function as a thioredoxin reductase, glutathione reductase, and glutaredoxin (43). Despite its low concentration in the liver, TRXR3 plays an essential role in the antioxidant system along with TRXR1 and TRXR2, besides their involvement in cell proliferation and redox-regulated signal cascades (44). Higher expression of *GPX2* and *GPX3* was observed in both disease groups when compared to HOC. GPX2 protein is present in the gastrointestinal tract, including the liver (45) and, therefore, may play a role in disease pathogenesis. However, GPX3 is secreted into the circulation (45), so any role it plays in NAFLD progression will be indirect and remote from the liver. Combined, molecular studies utilising mouse models of selenoprotein deficiency indicate that the effects on liver function cannot be generalised to all selenoproteins, as some are crucial while others can be less needed in hepatic physiology, and the specific role of each one should be considered. Absence of a clear pattern for the regulation of human hepatic selenoprotein expression between disease and control groups, as we herein report, reinforces this variability.

Other genes highly involved in the antioxidant response are *OPLAH*, which encodes 5-oxoprolinase, and *SOD1*, which encodes for Cu/Zn-superoxide dismutase 1. OPLAH is part of the glutathione cycle, responsible for converting 5-oxoproline, a degradation product of glutathione, into glutamate. Low expression of *OPLAH*, as seen in both steatosis and NASH, is associated with accumulation of 5-oxiproline and increased oxidative stress in *in vivo* and *in vitro* studies (46–48). Likewise, SOD1 is a key component of the cellular responses to oxidative stress, dismutating superoxides into hydrogen peroxide and molecular oxygen. The downregulation of *SOD1* in NASH that we uncovered in our analysis is consonant with animal model results, and suggests a profound implication of this gene in disease pathogenesis. Mice lacking the gene for SOD1 are considered an animal model to study hepatocellular carcinoma development following NASH, as they spontaneously present in their hepatocytes high oxidative stress, impaired very-low-density lipoprotein (VLDL) secretion that leads to lipid accumulation, necroptosis and inflammation (49). Consistent with this is the finding that *CCL2*, a proinflammatory chemokine, was increased in NASH compared with both control groups. CCL2 is known to be associated with development of inflammation and recruitment of monocytes in NASH (50). It is also compelling that, as NASH and NAFLD are risk factors for the development of hepatocellular carcinoma, the *JUN* proto-oncogene was also found to be upregulated in our analysis of NASH samples. *JUN* encodes for the c-Jun transcription factor subunit of the AP-1 complex (51) that participates in inflammatory responses to several types of cancer (52).

We found that *SCLY* expression was lower in NASH samples when compared to HC. *SCLY* encodes the enzyme Sec lyase (SCLY), that de-selenates organic Sec from dietary sources, the transsulphuration pathway or selenoprotein degradation (53) to provide selenide for selenoprotein synthesis. Hence, SCLY is at the intersection between degradation and biosynthesis of selenoproteins, allowing for recycling of selenium and thus may control selenium distribution (54). Interestingly, mice lacking SCLY develop hepatic steatosis (55), which is aggravated by selenium deficiency (55) and not restored by selenium supplementation (56); it is unknown if this mouse model develops NASH as it ages. The hepatic steatosis presented by the homozygous *SCLY* knockout mouse model informs that downregulation of *SCLY* may also be implicated in NAFLD development in humans. In addition, we found higher expression of *SEPHS2* in steatotic livers compared to HOC. *SEPHS2* is involved in inorganic selenite assimilation for selenoprotein synthesis. This gene was reported to be highly expressed in the rodent liver (57, 58). As both *SCLY* and *SEPHS2* play a role in providing selenium from different sources to selenoprotein synthesis, it is possible that either a shift in or redistribution of selenium sources for selenoprotein synthesis occurs in NAFLD livers.

The role of SCLY in selenoprotein degradation has been understudied, however its participation in the trans-selenation pathway is better understood. The trans-selenation pathway sequentially uses the methionine cycle enzymes and the transsulphuration pathway acting upon methionine to metabolise organic selenocompounds, particularly selenomethionine. The reactions of the methionine cycle are catalysed by methionine synthase (MS, encoded by the gene *MTR*) and methylenetetrahydrofolate reductase (MTHFR), while cystathionine beta-synthase (CBS) and cystathionine gamma-lyase (CTH) participate in the trans-selenation pathway to generate Sec as the final product, which serves as a specific substrate for SCLY (59). In addition to a lower expression of *SCLY*, our analysis uncovered that, overall, *MTHFR* and *CBS* were also less expressed in NAFLD, suggesting that methionine/selenomethionine metabolic capacity may be reduced in the disease. In alignment with our findings, a mouse model of fatty liver disease also presented with diminished CBS and transsulphuration metabolites, which negatively impacts glutathione metabolism (60), thus corroborating the critical participation of these selenium-related pathways in NAFLD pathogenesis. We speculate that the reduction in trans-selenation activity is either caused by diminished selenomethionine availability to be metabolised in NAFLD hepatocytes or a consequence of dysfunctional methionine metabolism that impacts selenium metabolism as collateral damage. Nevertheless, the observed lower expression of *SCLY, MTHFR*, and *CBS* in the disease, is possibly indicative of a reduced transsulphuration capacity, which is required for the metabolisation of methionine and selenomethionine. This, combined with the higher expression of *SEPHS2*, strengthens the hypothesis of a shift in selenocompound utilisation during NAFLD pathogenesis to be tested in the future. Moreover, it suggests that a role for selenium metabolism beyond selenoproteins may persist in NAFLD's pathophysiology.

Ferroptosis is a unique non-apoptotic-programmed cell death pathway dependent on iron and lipid hydroperoxides. This pathway is characterised by the inhibition of the xc– system, responsible for Cys import, causing limited glutathione biosynthesis. Additionally, disturbed intracellular iron storage and PUFA-enriched phospholipids form the scenario required for ferroptosis (61). Given that GPX4 can reduce lipid peroxides when glutathione levels are decreased, this selenoprotein is a key negative regulator of this cell death pathway (62). Ferroptosis was reported as the initial cell death mechanism that triggers steatohepatitis (63). Changes in iron parameters, including increased serum ferritin and hepatic iron, have often been reported in association with NAFLD, although the evidence is conflicting (64), suggesting that any role for iron in NAFLD is likely to be the target of multiple regulatory processes, hence the importance of exploring this pathway in this study. Our analysis demonstrated that, compared to HC, *SLC11A2*, the gene encoding the importer, divalent metal transporter 1, was less expressed in NAFLD. Although not statistically significant, *SLC11A2* was also lower compared to HOC. Similarly, *SLC40A1*, the gene encoding the iron exporter ferroportin, was lower in steatosis and NASH than in HOC, and caeruloplasmin (*CP*) was decreased in NASH compared to both control groups. Ferroportin and CP act together to export cellular iron, ferroportin transporting ferrous iron through the membrane and CP catalysing oxidation to ferric iron, which is necessary for release (65). These findings suggest a contraction of hepatic iron metabolism in NAFLD, whereby iron uptake is being decreased, and the release of iron already within the cell is curtailed. This is consistent with the observed higher expression of genes encoding the ferritin subunits (*FTL* and *FTH1*) in the disease, suggesting an increased capacity for storage of iron already in the cell. Ferritin is regulated post-transcriptionally (66), with protein levels being more responsive to iron than mRNA levels, so the decrease in transcript observed in these datasets suggests a chronic response.

Expression of *ACSL4*, an acyl-CoA synthetase, is higher in NASH compared both to HC and HOC. *ACSL4* is a known stimulator of iron-associated cell death (67), being involved in the synthesis of polyunsaturated fatty acids, which are conducive to peroxidation. In contrast, *ACSL5* was less expressed in NASH compared to HC. ACSL5 has been associated with apoptotic cell death in the gut and steatotic liver, and decreased ACSL5 with reduced sensitivity to cell death in HepG2 cells (68, 69). *ACSL6* is more highly expressed in NASH than HOC, although it has previously been reported in several cell lines, including the hepatoma cell line HepG2, not to be associated with ferroptosis (67). Increases in acyl-CoA synthetases are consistent with our previous findings in AML12 mouse hepatocytes that lipid accumulation occurs within 16 hours of iron loading and continues to increase over the subsequent 8 hours (70). However, the mechanism behind differential regulation of three *ACSL* isoforms, all of which are associated with cell death pathways, remains unclear. ACSL4, ACSL5, and ACSL6 have substrate preferences for long-chain polyunsaturated fatty acids (C20 and C22), although ACSL6 is more specific for docosahexaenoic acid (DHA; C22:6) (71–73) and its hepatic expression is lower (36). However, we cannot rule out that higher expression of *ACSL4* and *ACSL6* may simply be associated with a general upregulation of lipid-related gene expression in NAFLD (74).

Gene expression of autophagy-like proteins was higher in the disease states: *ATG7* was higher in NASH in comparison to HC, and ATG5 was higher in steatosis when compared to HOC. These proteins participate in a ubiquitin-like pathway which is a precursor to phagophore formation (75). Maturation of phagophores into autophagosomes requires *MAP1LC3* (76), of which the alpha form is more highly expressed and the gamma form less expressed in NAFLD than HOC. Together, these observations suggest induction of autophagy to remove damaged cell components, although potentially not induction of ferroptosis. Whilst ferritin degradation may occur *via* autophagy (so-called ferritinophagy) to release iron that then induces ferroptosis (77), it is unclear why ferritin transcripts would be more highly expressed under a ferroptotic phenotype. Autophagic pathways are a component of many degradation pathways and may not necessarily lead to cell death; hence, the upregulation of ferritin may allow the cell to sequester iron and repair oxidative or inflammatory damage without committing to ferroptotic cell death. Confirmation of this hypothesis would require further investigation.

The lower expression of plasminogen (*PLG*) and higher expression of *SERPINE1*, which encodes plasminogen activator inhibitor-1 (PAI-1) in NAFLD may be an indicator of the presence of a prothrombotic state in the disease. PAI-1 inhibits the tissue plasminogen activator, resulting in a decreased conversion of plasminogen into active plasmin, which is crucial for degradation of fibrin clots (78). Animal models show that PAI-1 is associated with fat accumulation in the liver (79) and progression of inflammation and hepatic fibrosis (80). Given the association between PAI-1 and endothelial dysfunction (81, 82), Ciavarella et al. (83) emphasised that PAI-1 plays a critical role in the pathogenesis of a prothrombotic state in NAFLD, which links the disease with an increased risk for cardiovascular events.

Overall, our findings indicate a larger difference between NASH and HC than when NASH was compared to either HOC or steatosis. Although this does not apply to all the genes of interest, this observation supports the idea of a progressive change in gene expression across the different stages of the disease. Surprisingly, no differences between HC and steatosis were observed, while steatosis presented some significant differences when compared with HOC. Although this should be further investigated, we hypothesise that this is a product of the criteria we applied, given that only two datasets had data for the comparison between HOC and steatosis, and therefore the log fold changes are more consistent in these two than in the four datasets used for the comparisons with HC.

Limitations of whole -omics analysis include the potential to miss subtle, yet biologically relevant, pathway alterations. This is particularly pertinent in trace element homeostasis, where slight alterations in transcript, protein or metabolite levels may have large downstream effects. A key strength of this study relies on the investigation of specific pivotal pathways, thus enabling the exploration of molecular mechanisms that are commonly an oversight in global exploratory studies. Given that this study concerns a bioinformatic analysis of a limited number of genes and is of an exploratory nature, a stringent adjustment method (Benjamini-Hochberg) was chosen to minimise false-positive over false-negative results. Comparing liver samples between steatosis, NASH, and controls with and without obesity advances the understanding of gene expression changes associated with the progressive risk of NAFLD, even though this comparison has been limited by the inclusion of only two studies that reported data for HC and HOC. Small sample sizes in omics studies often hinder the exploration of modest variations in gene expression in complex diseases such as NAFLD. Thus, the comparison of multiple studies as performed in this analysis allows for larger sample size and increased utility of these data, ultimately supporting translation of research findings into practise. Nonetheless, this approach imposes some limitations that should be acknowledged. As our bioinformatics analysis used publicly available data, we were limited by the lack of information on the subjects' individual characteristics, including demographics (age and ethnicity) and clinical history which may have shed some additional insight into the variability between datasets. The inclusion of this additional phenotypic information for the datasets available in the repositories would allow more complex analysis to facilitate data interpretation and increase the utility of these data more broadly. The lack of dietary data precludes identifying potential differences in selenium intake, which could explain some differences seen across different studies. Furthermore, studies included in our analysis used different scoring systems for histopathologic classification of liver lesions used by the selected studies. Although this can lead to changes in the discrimination between steatosis and NASH, the scoring methods applied can appropriately separate controls and disease. Also, the included datasets obtained control samples from different situations, such as major surgeries or liver tissue bank (which commonly receives samples from subjects involved in motor vehicle accidents), which may be implicated in different underlying physiological processes. This study brings insight into the role selenium plays in NAFLD. Still, given its exploratory nature, future research is needed to validate our findings, for example, through qPCR or Nanostring technology. A combined proteomics approach would add further information as to whether our findings persist at the protein level.

## Conclusions

Our study brings findings from a preliminary analysis that shed light on how selenium may be involved in the regulation of pathological mechanisms in NAFLD. Our findings suggest that the NAFLD liver may have lower selenium levels than a disease-free liver, and further studies targeting *TXNRD3* and *SCLY* along with enzymes involved in the transsulphuration pathway (particularly *MTHFR* and *CBS*) may elucidate the pathophysiological role of selenium metabolism in NAFLD. Furthermore, our study provides evidence for a link between selenium and iron metabolism, which is known to be disrupted in NAFLD, and may contribute to cell death *via* ferroptosis, ultimately leading to cirrhosis.

## Data Availability Statement

Publicly available datasets were analysed in this study. This data can be found here: Repositories: GEO and ArrayExpress. Accession Numbers: GSE126848, E-MEXP-3291, GSE61260, GSE63067, and GSE48452.

## Ethics Statement

Ethical review and approval was not required for the study on human participants in accordance with the local legislation and institutional requirements. The patients/participants provided their written informed consent to participate in this study.

## Author Contributions

KD and BC: study conceptualisation and writing—original draft. KD: data curation and formal analysis. LS, RG, and BC: writing—review and editing. BC: supervision. All authors contributed to the article and approved the submitted version.

## Funding

This study was supported by Hawaii Community Foundation Research Grant 20ADVC-102166, National Institute of Diabetes and Digestive and Kidney Diseases Grant R01DK128390-01, and Administrative Supplement 3R01DK047320-23S1 (LS); Monash Graduate Scholarship and Monash International Tuition Scholarship (KD).

## Conflict of Interest

The authors declare that the research was conducted in the absence of any commercial or financial relationships that could be construed as a potential conflict of interest.

## Publisher's Note

All claims expressed in this article are solely those of the authors and do not necessarily represent those of their affiliated organizations, or those of the publisher, the editors and the reviewers. Any product that may be evaluated in this article, or claim that may be made by its manufacturer, is not guaranteed or endorsed by the publisher.

## Abbreviations

BMI: body mass index
CBS: cystathionine beta-synthase
CP: caeruloplasmin
CTH: cystathionine gamma-lyase
Cys: cysteine
DHA: docosahexaenoic acid
DIO: iodothyronine deiodinase
GEO: Gene Expression Omnibus
GO: gene ontology
GPX: glutathione peroxidase
HC: normal weight healthy control
HOC: healthy obese control
INS: insulin
KEGG: Kyoto Encyclopaedia of Genes and Genomes
MS: methionine synthase
MSRB1: methionine sulphoxide reductase B1
MTHFR: methylenetetrahydrofolate reductase
NAFLD: non-alcoholic fatty liver disease
NAS: NAFLD activity score
NASH: non-alcoholic steatohepatitis
PAI-1: plasminogen activator inhibitor-1
PCA: principle component analysis
PLG: plasminogen
RMA: robust multi-array averaging
ROS: reactive oxygen species
SAF: Steatosis-Activity-Fibrosis
SCLY: selenocysteine lyase
Sec: selenocysteine
SELENOP: selenoprotein P
SEPHS2: selenophosphate synthetase 2
TRXR: thioredoxin reductase
TSTD1: Thiosulfate Sulfurtransferase Like Domain Containing 1
UGT1A6: UDP Glucuronosyltransferase Family 1 Member A6
VLDL: very-low-density lipoprotein.
