# Supplementary material for: Selenotranscriptome Network in Non-alcoholic Fatty Liver Disease
Source: Front Nutr. 2021 Nov 17;8:744825. doi: 10.3389/fnut.2021.744825 (PMC8635790; doi:10.3389/fnut.2021.744825)
Supplement: Supplementary file 3 [file Image_1.pdf]

Supplementary Material

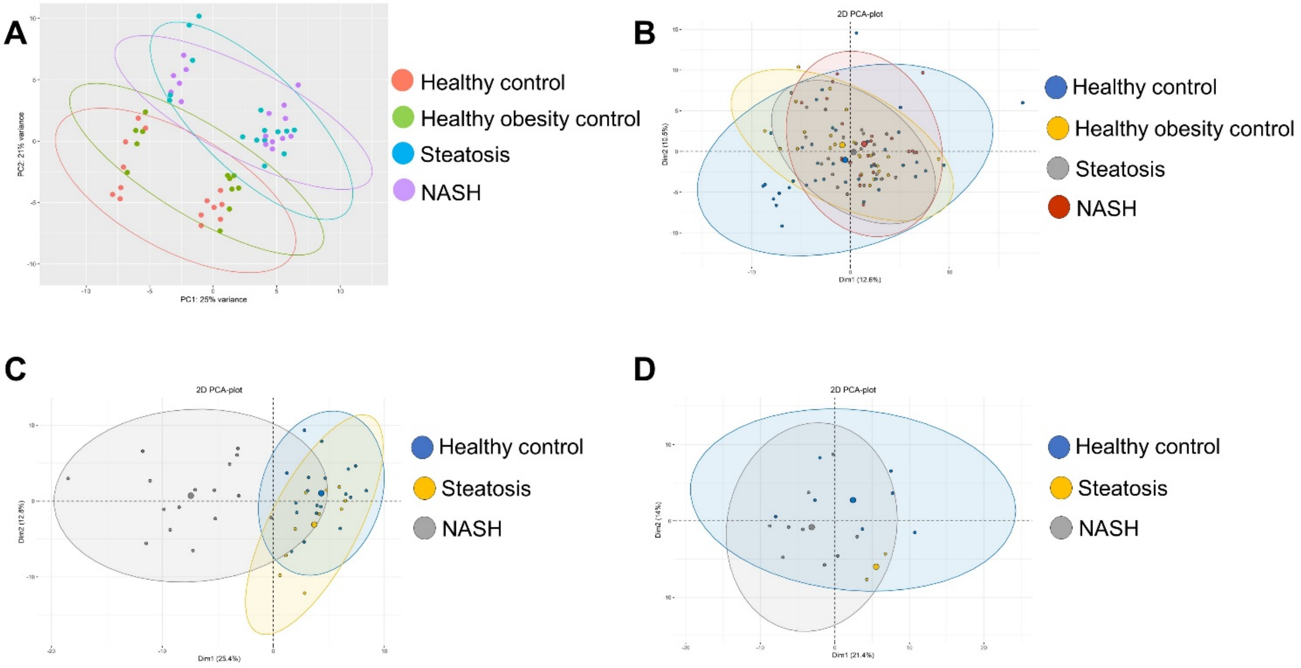

Supplementary Figure 1. PCA plots for: A) Suppli et al. [29], B) Horvath et al. [31], C) Lake et al. [30], D) Frades et al. [25].
